# Parent-Child Play and the Emergence of Externalizing and Internalizing Behavior Problems in Childhood: A Systematic Review

**DOI:** 10.3389/fpsyg.2022.822394

**Published:** 2022-05-02

**Authors:** Mirjam Schneider, Irina Falkenberg, Philipp Berger

**Affiliations:** ^1^Department of Psychiatry and Psychotherapy, Philipps-University Marburg, Marburg, Germany; ^2^Center for Mind, Brain and Behavior (CMBB), University of Marburg and Justus Liebig University Giessen, Marburg, Germany; ^3^Department of Neuropsychology, Max Planck Institute for Human Cognitive and Brain Sciences, Leipzig, Germany; ^4^Research Group Milestones of Early Cognitive Development, Max Planck Institute for Human Cognitive and Brain Sciences, Leipzig, Germany

**Keywords:** play, parental playfulness, behavioral adjustment, externalizing and internalizing behavior problems, systematic review

## Abstract

It has widely been accepted that play has a major role in human development. The play situation is considered a save and controlled space in which children can learn to express their problems and to regulate their emotions, thus promoting emotional and behavioral adjustment. In early childhood, this process is thought to emerge in close interaction with caregivers. Parent-child play is thus viewed as an ideal window for parents to connect with their children and to support them in their social-emotional development. In this preregistered systematic review, we sought to integrate evidence from developmental and clinical psychology to shed more light on the role of parents in the relationship between parent-child play and children's behavioral adjustment as expressed in internalizing or externalizing behavior. Our review revealed that increased harsh control during play interactions as well as a lack of parental responsiveness, warmth and sensitivity were found to be associated with increased behavioral problems. Yet, no protective effect of warmth or responsiveness could be found in the context of risk groups. Moreover, the included studies indicated that positive affect expressed by parents during parent-child play was associated with fewer behavior problems in children, while negative affect was associated with more behavior problems. In general, this review revealed that quality and quantity of playful parent-child interactions were reduced in children with behavioral problems of both domains compared to children without behavioral problems. These findings illustrate the important role of parental characteristics during play interactions and their possible impact on children's behavioral adjustment.

## Introduction

It is widely accepted that *play* has an important role in human development and is particularly influential in early childhood (Whitebread et al., 2017). The *play-situation* involves children and parents in highly interactive and intimate situations that may pose an important setting in which children gain social and self-regulation skills fundamental to behavioral adjustment (Axline, 1969; Erikson, 1993). Thus, the play-situation is also commonly used in therapeutic settings with children and a broad range of interventions focusing on parent-child play have been established to improve behavioral adjustment in children, i.e., their ability to flexibly adjust their own behavior to changing social and emotional situations. To measure this behavioral adjustment, it is sensible to observe and measure externalizing (e.g., aggressiveness, impulsivity) and internalizing (e.g., withdrawal, anxiety) problem behavior. As a prominent example, during feedback-based Parent-Child Interaction Therapy (PCIT; Eyberg et al., 1995) parents are instructed how to adequately react to their child's behavior in a play situation to create a more positive interaction and improve the parent-child relationship. Several studies implementing PCIT have shown positive short-term and long-term effects on behavior problems in children as well as parental stress (see Thomas et al., 2017 for a meta-analysis), indicating that parenting behavior during parent-child play might be considered an important mechanism in the emergence of maladaptive behavior. While the suggestion that parent-child play in a therapeutical setting has a substantial relation to their social-emotional development was corroborated by a wide range of empirical research, the particular role of parents and caregivers on this relation and outside the therapeutical context has not been systematically investigated. In this preregistered review, we sought to integrate evidence from developmental and clinical psychology to shed more light on the role of parents in the relationship between everyday parent-child play interactions and children's social-emotional development. In particular, we will investigate if and how parental variables in parent-child play show an impact on children's externalizing and internalizing behavior problems.

### Play and Playfulness Across Development

Children spend a large part of their day playing, quietly or loudly, whether alone or in a group, with toys or without. In more advanced pretend play, children even mimic adults or create their own fantasy world. In doing so, play not only leads to immediate amusement and enjoyment from the perspective of the child, but may thereby also contribute significantly to child development and health (Lester and Russell, 2010; Amodia-Bidakowska et al., 2020). In his model of psychosocial developmental stages, Erikson gives a firm place to the *play age* as the third of his eight postulated stages of human development across the lifespan (Erikson, 1993). The model suggests that at this developmental stage in early childhood, children begin to take the initiative to interact with other children and to playfully explore and discover their interpersonal and social skills. This initiative, however, is proposed to be contrasted with guilt: as initiative increases, the child experiences more criticism or control from others, mostly parents and caregivers, which according to Erikson leads to feelings of guilt, but which in turn might also be important for the development of self-control and behavioral adjustment. Thus, throughout early child development, a balance should be established between initiative and guilt, such that important skills can be formed in interaction with other individuals, especially with parents and caregivers. In this influential description of the nature and purpose of children's play it was suggested that play has a particular fundamental role in the development of social-emotional skills, moral sense, and self-regulation (Erikson, 1993). This idea is also reflected in recent empirical studies indicating that higher play quality is associated with better self-regulation (Cabrera et al., 2017; Stgeorge and Freeman, 2017), social competence (Pettit et al., 1998), and cognitive development (Mermelshtine and Barnes, 2016) in children. Moreover, children have been suggested to use the rather controlled and save environment in the play situation to express their problems and regulate their emotions (Axline, 1969; Landreth, 2002).

However, besides visible *play behaviors*, play is commonly defined as an intrinsically motivated, process-oriented, freely chosen, and enjoyable activity (Johnson et al., 2005) that comprises behavioral and internal aspects. The internal basis of play, referred to as *playfulness*, has been found to be relatively stable, even until later adulthood (Lieberman, 1965; Singer and Singer, 1978; Singer et al., 1980; Barnett, 1990). In this context, *playfulness* is understood as a trait, contributing to the ability of children and adults to create a playful world even in the most dull environment (Barnett, 1990). While the majority of studies has characterized the nature of children's play, recently, an increasing amount of research has focused on characterizing *adult* play and playfulness as well. Barnett (2018) formed a rather detailed definition of playfulness in adults, characterizing playful adults as “*funny, humorous, spontaneous, unpredictable, impulsive, active, energetic, adventurous, sociable, outgoing, cheerful, and happy, [...] likely to manifest playful behavior by joking, teasing, clowning, and acting silly*” (Barnett, 2007, p. 955). Thus, across development, playfulness might be described to involve (1) developing pleasant, funny, or creative thoughts and ideas (2) without significant concern for pleasing others (e.g., social expectations) or for the consequences of one's actions, (3) often visible as good-natured humor (Barnett, 2018). In their function as caregivers and in interacting with children, adults frequently—and sometimes unconsciously—make use of their playfulness. Particularly, in parent-child interactions, parents' playfulness is manifested in the parents' ability to reframe different situations with their child in a playful manner, turn harsh situations into fun, and act in a flexible, humoristic, creative manner in times of stress (Barnett, 1990; Shorer et al., 2019; Levavi et al., 2020). Research on parental playfulness typically uses observation of free or structured parent-child play, and self-rating questionnaires (Amodia-Bidakowska et al., 2020). In general, these observations have revealed close associations with parenting behavior, such that more parental warmth and acceptance, and less permissiveness and rejection seem to be reflected in high levels of parental playfulness observed in parent-child play (Lucassen et al., 2011; Kim and Shin, 2014; Han and Lee, 2015; Waller et al., 2015).

### Behavioral Adjustment in Children

This review focuses on the impact of parent-child play on children's behavioral adjustment in particular. Behavioral adjustment describes the ability of children to flexibly adjust their own behavior to changing social and emotional situations. This is operationalized best using qualitative or quantitative measures of behavioral problems: the fewer behavioral problems are detected, the better the behavioral adjustment. A common distinction is made between externalizing and internalizing behavioral problems (Achenbach, 1966, 1978; Eisenberg et al., 2001). Externalizing problematic behavior is openly displayed to the external environment, e.g., aggression, impulsivity, hyperactivity, or delinquency (Hinshaw, 1987; Shaw and Gilliom, 2000; Eisenberg et al., 2001; Liu, 2004). Internalizing behavior problems in turn primarily affect the internal world of a person. Examples are reclusiveness, somatic pain as well as anxious, inhibited, or depressive behavior (Eisenberg et al., 2001; Liu, 2004; Liu et al., 2011). Such behavioral problems during childhood and adolescence are frequent and certain combinations of these peculiarities are early warning signs or risk factors for psychiatric illnesses (Achenbach and Ruffle, 2000; Liu, 2004). Externalizing behavior problems are considered the most common type of maladjustment and implicate an increased risk for psychosocial problems and psychiatric illnesses later in life (Campbell et al., 2000). Against this background, it is important to investigate factors that might have a positive impact on behavioral adjustment, e.g., during parent-child play as this is one of the most prevalent types of parent-child interactions in early childhood.

### The Association of Play-Related Parenting Behavior With Behavioral Adjustment

The relationship between parenting behavior in general with child development has been intensely researched in psychology and beyond (Belsky and De Haan, 2011). These studies have revealed a complex picture showing that both parenting behavior, as well as individual facets, such as parental sensitivity and warmth, show distinct and meaningful associations to child development (Deater-Deckard, 2000; Rohner and Britner, 2002; Pinquart, 2016, 2017). A common perspective in this context is the dimensional approach to describe parental behavior according to the two dimensions warmth/responsiveness and control/demandingness. High warmth/responsiveness includes aspects like acceptance, support, and sensitivity whereas low scores on this dimension are observable as rejection, hostility, or insensibility (Maccoby and Martin, 1983). Control/demandingness on the other hand can be divided into behavior/authoritative control, harsh/authoritarian control (verbal and physical punishment as well as intrusiveness), and psychological control (e.g., manipulation) (Barber, 1996; Pinquart, 2017). As one of the most important associations between these behavioral characteristics and child development, it could be found that parenting behavior is particularly related to behavioral adjustment in children (e.g., Haapasalo and Tremblay, 1994; Steinberg et al., 1994; Pettit et al., 1997; Li et al., 2000; Waller et al., 2015; Pinquart, 2017), which is observable as externalizing or internalizing behavior problems as described above. In a meta-analysis by Pinquart (2017), it could be shown that certain dimensions of parenting behavior, such as harsh control, psychological control, as well as permissive and neglectful parenting were strongly associated to high levels of externalizing behavior problems in children. Similarly, parenting behavior, such as parental hostility and rejection and abusive parenting, has been connected to the emergence of internalizing behavior problems in children in a range of studies (McLeod et al., 2007a,b; Yap and Jorm, 2015). Additionally, positive parental behavior, such as high warmth and behavior control, shows small to moderate associations to less behavior problems in children (Pettit et al., 1997; Hoeve et al., 2009; Pinquart, 2017). As mentioned above, these positive parenting behaviors also seem to be reflected in high levels of parental playfulness observed in parent-child play (Lucassen et al., 2011; Kim and Shin, 2014; Han and Lee, 2015; Waller et al., 2015) and a major component of PCIT is to establish warmth and confidence in parenting behavior during parent-child play, using the play-situation as a secure and safe environment for changes in the parent-child relationship (PCIT; Eyberg et al., 1995).

Relatedly, observing play situations and everyday activities, Shorer et al. (2019) found direct evidence that parental playfulness, the caregivers' disposition to engage in playful interactions with their child, is associated with improved emotion regulation in early childhood. Further, in a study by Menashe-Grinberg and Atzaba-Poria (2017), the relationship between playful parent-child interactions and child negativity was examined, which included both oppositional behavior and negative affect (such as anger, sadness, and fear). The results showed that higher playfulness in parents was generally associated with lower child negativity. In sum, these findings from previous studies suggest that parental playfulness and parent-child play may be an important factor in the emergence of internalizing and externalizing behavior problems in childhood. However, although the play-situation is frequently used in (i) clinical applications that attempt to improve behavioral adjustment and (ii) in observational research on the influence of parenting behavior on behavioral adjustment, it remains unclear which factors in parent-child play may be of particular importance.

### The Current Study

In this preregistered systematic review, we sought to integrate research from developmental and clinical psychology to shed more light on the role of parent-child play in the emergence of behavioral adjustment. Specifically, we asked how parental variables during parent-child play in early childhood (up to the age of 4 years) are related to the emergence of behavioral adjustment and the onset of externalizing and internalizing behavioral problems. We chose to investigate parent-child play during early childhood in particular since this is a period of crucial sensitivity to experiences that promote development (Marshall and Kenney, 2009; Britto et al., 2017). Moreover, the recent review article on father-child play by Amodia-Bidakowska et al. (2020) shows that parent-child play in this age is critical for later developmental outcomes as children's self-regulation, aggression, or cognitive skills. As described above, there is several literature on the effect of parent-child play on developmental outcomes as e.g., self-regulation as well as several studies investigating the effects of parenting variables (especially along the two axes control and warmth described by Maccoby and Martin, 1983) on children's behavioral adjustment. Based on findings from these two related approaches, we expected to find a variety of studies in our systematic literature search indicating that higher levels of positive parenting variables during parent-child play also correlate with better behavioral adjustment and thus reduced quantity and quality of externalizing and internalizing behavioral problems in childhood and adolescence.

## Methods

The present work follows the NIRO guidelines for non-interventional, reproducible and open systematic reviews (Topor et al., 2020) and was preregistered according to these specifications on the OSF platform (open science framework, https://osf.io/2jbst). In the preregistration protocol, we pre-specified how the systematic literature search will be conducted and according to which inclusion and exclusion criteria studies were or were not considered for this review.

### Systematic Literature Search

A systematic literature search in the database PsycINFO (American Psychological Association) was performed in April 2021, using the boolean search string: [TI(parental playfulness OR mother-child play OR father-child play OR parent-child play OR parent-child interaction OR mother-child interaction OR father-child interaction OR parental play) OR AB(parental playfulness OR mother-child play OR father-child play OR parent-child play OR parent-child interaction OR mother-child interaction OR father-child interaction OR parental play)] AND [TI(internal^*^ OR external^*^ OR behavior problem^*^ OR anxiety OR depress^*^ OR conduct OR psychopathology) OR AB(internal^*^ OR external^*^ OR behavior problem^*^ OR anxiety OR depress^*^ *OR conduct OR psychopathology)] NOT play therapy NOT autism*.

**Figure 1:**
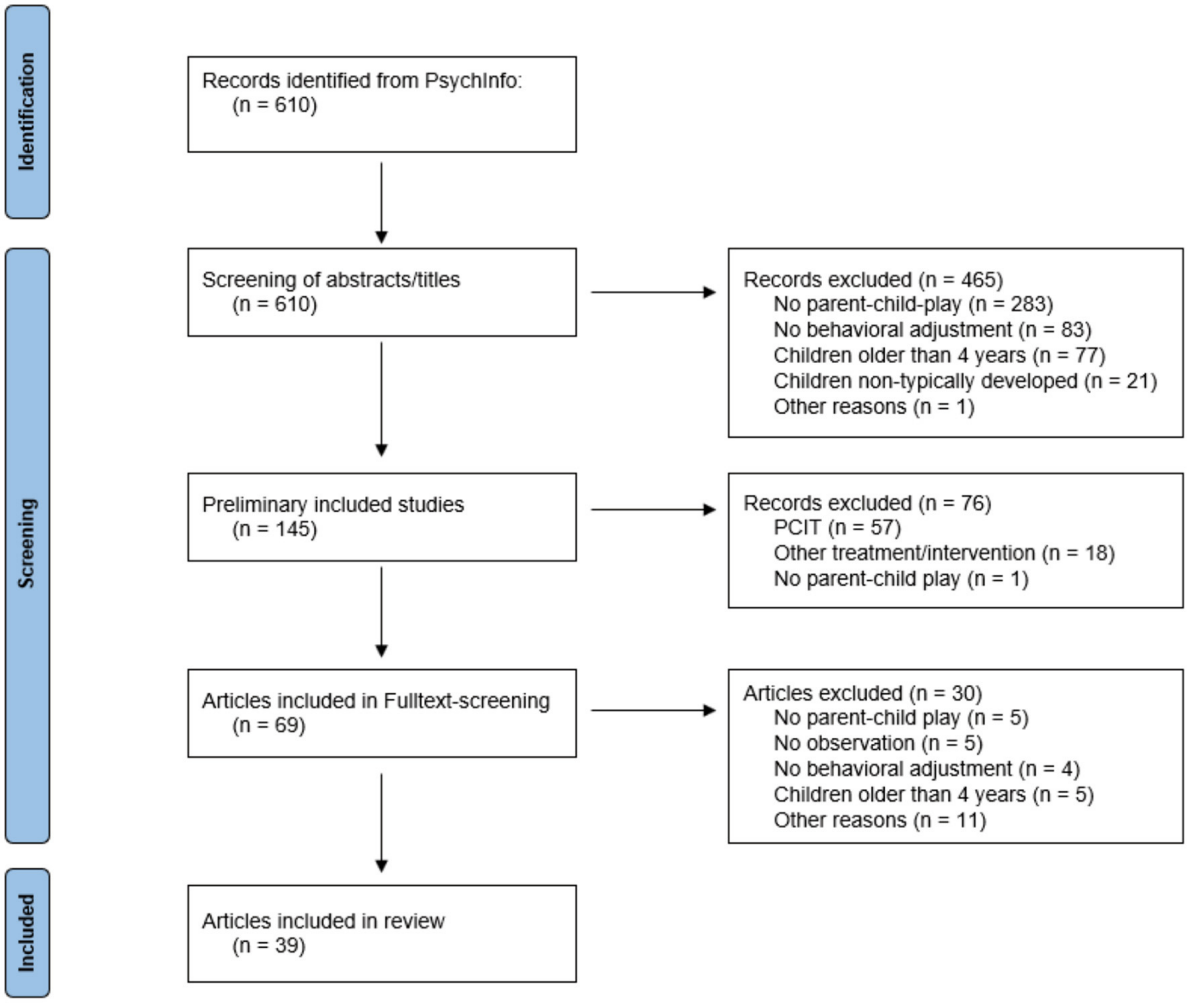
Flow chart describing literature search with number of included and excluded records.

We explicitly excluded therapeutic interventions and children with autism (or other developmental disorders) as those aspects go beyond the scope of our review, in which we focus on everyday parent-child play and healthy children. Additionally, several limiters were set to restrict literature search to (1) publications in English, (2) publications in peer-reviewed journals, (3) publications about children in preschool age or younger (≤ 4 years). This search resulted in the identification of 610 articles, which were then screened for eligibility (see Figure 1 for a flow chart on study search and selection).

### Study Inclusion and Exclusion

After initial selection of studies, we applied title and abstract screening with python-based ASReview (van de Schoot et al., 2020), using our preregistered exclusion criteria. In particular, records were excluded that: (1) did not operationalize parental playfulness or parent-child play (*N* = 283), (2) did not research children's behavioral adjustment as dependent variable (*N* = 83), (3) did not include children younger than 4 years old (*N* = 77), (4) did not include typically developing children (*N* = 21), (5) did not fit our research question otherwise [*N* = 1 (study protocol)]. The classification was conducted by the first author, and two additional blind reviewers rated randomly selected 25% of the abstracts for eligibility (Cronbach's *a*= 0.79). The initial screening resulted in n = 145 studies that were deemed eligible according to title and abstracts. As an intermediate step, we decided to define an additional exclusion criterion, namely the exclusion of studies that imply Parent-Child Interaction Therapy (PCIT; *N* = 57) or similar treatments or interventions (*N* = 18). Treatments and interventions were not the focus of our research question and constitute a whole research topic on their own that is beyond the scope of this review. Furthermore, another record that did not operationalize parental playfulness or parent-child play was identified and excluded (*N* = 1). This intermediate screening step resulted in N = 69 studies to be included in full-text screening. The remaining articles then underwent full-text screening, applying our preregistered inclusion and exclusion criteria. Thus, studies were excluded that (1) did not explicitly measure parental playfulness or parent-child play as an independent variable (*N* = 5). Additionally, (2) we only included studies that operationalized parental playfulness or parent-child-play as an observer-rated measure in parent-child-interactions. Studies that consider playfulness more broadly as a trait or did not measure it in a concrete playing situation were excluded (*N* = 5). (3) Studies that did not measure behavioral adjustment as a dependent variable (*N* = 4) were excluded. While standardized parental rating scales, such as the Child Behavior Checklist (CBCL; Achenbach and Ruffle, 2000), have been developed to assess behavior problems in early childhood, we additionally included clinical manifestations of maladaptive behavior, such as ADHD or conduct disorders for the externalizing, and anxiety or depression for the internalizing domain. Furthermore, 4) we identified an additional number of studies that did not include children younger than 4 years old (*N* = 5) as well as 5) studies not fitting our research question otherwise (*N* = 8). No studies were excluded due to severe methodological flaws. This procedure resulted in 39 studies that were included in our review (see Figure 1 for an overview of study selection, see Table 1).

**Table 1:** Characteristics of studies included in the narrative syntheses (*k* = 39) with information on study design, sample characteristics (parent's gender, number of parent-child dyads, country of acquisition), independent variable(s) and dependent variable.

**References**	**Study design**		**Sample characteristics**			**Independent variable(s) (during parent-child play)**	**Dependent variable (behavior problems)**
			**Parent**	**Number of parent-child dyads**	**Country of acquisition**		
Mence et al. (2014)	Cross-sectional	Quantitative	Both	82	Australia	Control/discipline	Externalizing
Janßen et al. (2019)	Cross-sectional	Quantitative	Mothers	80	Germany	Control/discipline	Boths
Hughes and Ensor (2007)	Longitudinal	Quantitative	Mothers	120	GB	Control/discipline, warmth	Boths
Lee and Bates (1985)	Longitudinal	Quantitative	Mothers	111	USA	Control/discipline	Externalizing
Heller and Baker (2000)	Longitudinal	Quantitative	Mothers	120	USA	Control/discipline	Externalizing
Webster-Stratton and Eyberg (1982)	Cross-sectional	Quantitative	Mothers	35	USA	Control/discipline	Boths
Smeekens et al. (2007)	Longitudinal	Quantitative	Both	116	Netherlands	Control/discipline	Externalizing
Dubois-Comtois et al. (2021)	Cross-sectional	Quantitative	Fathers	81	Canada	Control/discipline	Boths
Williams et al. (2012)	Cross-sectional	Quantitative	Both	161	USA	Warmth	Internalizing
Lorber and Egeland (2011)	Longitudinal	Quantitative	Mothers	267	USA	Warmth	Boths
Scaramella et al. (2008)	Longitudinal	Quantitative	Mothers	40	USA	Warmth	Externalizing
Schrock and Woodruff-Borden (2010)	Cross-sectional	Quantitative	Both	158	USA	Warmth	Internalizing
Jambon et al. (2019)	Longitudinal	Quantitative	Mothers	424	Canada	Warmth	Externalizing
Murphy et al. (2017)	Longitudinal	Quantitative	Both	71	USA	Warmth	Externalizing
Schmid et al. (2011)	Longitudinal	Quantitative	Mothers	314	Germany	Warmth	Internalizing
Ambrose and Menna (2013)	Cross-sectional	Quantitative	Mothers	73	Canada	Warmth	Externalizing
Sirois et al. (2019)	Longitudinal	Quantitative	Mothers	86	Canada	Warmth	Boths
Kok et al. (2013)	Longitudinal	Quantitative	Mothers	886 and 935	Netherlands and USA	Sensitivity	Internalizing
van der Voort et al. (2014)	Longitudinal	Quantitative	Mothers	146	Netherlands	Sensitivity	Internalizing
NICHD Early Child Care Research Network (2004)	Longitudinal	Quantitative	Mothers	1,364	USA	Sensitivity	Boths
Morrell and Murray (2003)	Longitudinal	Quantitative	Mothers	59	GB	Sensitivity	Externalizing
Denham et al. (1991)	Cross-sectional	Quantitative	Mothers	47	USA	Affect, control/discipline	Boths
Robinson et al. (2009)	Cross-sectional	Quantitative	Mothers	123	USA	Affect	Internalizing
LaFrenière and Dumas (1992)	Cross-sectional	Quantitative	Mothers	126	Canada	Affect	Internalizing
Dollberg et al. (2006)	Cross-sectional	Quantitative	Mothers	79	Israel	Affect, sensitivity	Internalizing
Endendijk et al. (2017)	Longitudinal	Quantitative	Mothers	96	Netherlands	Affect	Boths
Dumas et al. (1989)	Cross-sectional	Quantitative	Mothers	51	Canada	Affect	Boths
Lang et al. (1996)	Cross-sectional	Quantitative	Mothers	29	USA	Affect	Boths
Colonnesi et al. (2019)	Longitudinal	Quantitative	Both	104	Netherlands	Mind-mindedness	Boths
Lunkenheimer et al. (2013)	Longitudinal	Quantitative	Mothers	100	USA	Teaching	Boths
Urbain-Gauthier and Wendland (2017)	Cross-sectional	Quantitative	Mothers	150	Belgium	Quality of play, warmth	Externalizing
Landy and Menna (2001)	Cross-sectional	Qualitative	Mothers	60	Canada	Quality of play, warmth	Externalizing
Ahnert et al. (2017)	Cross-sectional	Quantitative	Both	103	Austria and Germany	Quality of play	Internalizing
Gardner (1994)	Cross-sectional	Quantitative	Mothers	39	GB	Quality of play	Externalizing
Gardner et al. (2003)	Longitudinal	Quantitative	Mothers	59	GB	Quality of play	Boths
Yates et al. (2010)	Longitudinal	Quantitative	Mothers	209	USA	Interaction quality in general	Boths
Dubois-Comtois et al. (2015)	Cross-sectional	Quantitative	Foster mothers	83	Canada	Interaction quality in general	Boths
Cao et al. (2020)	Longitudinal	Quantitative	Mothers	823–1,364	USA	Interaction quality in general	Boths
Gardner (1987)	Cross-sectional	Quantitative	Mothers	39	GB	Interaction quality in general	Externalizing

### Coding

Studies were identified by the above systematic literature search and, if applicable, coded for (1) sample characteristics, (2) study design (longitudinal vs. cross-sectional, qualitative vs. quantitative), (3) operationalization of parental variables or parent-child play, (4) operationalization of child behavioral adjustment, (5) limitations of the study. The detailed results of this coding procedure can be reviewed in the Table S1 (supplemental information at https://osf.io/arg8j/). A narrative synthesis of each area of research is presented below, identifying the main trends in findings. Note that due to the heterogeneity of study designs, measures, and populations it was not possible to perform a meta-analysis.

## Results

The literature search outlined above yielded 39 articles that met the preregistered inclusion criteria (see Table 1 for an overview). Contrary to our assumption in the preregistration, however, only a few of the articles directly investigated the quality of parent-child play in association with child behavioral adjustment (Gardner, 1994; Landy and Menna, 2001; Gardner et al., 2003; Ahnert et al., 2017; Urbain-Gauthier and Wendland, 2017) and no studies were found directly investigating parental playfulness. Instead, the majority of studies used parent-child play as a context for observing general parental and child interaction variables that were then examined for a connection with behavioral adjustment. From these studies, however, we can draw important information about the association between qualitative aspects of parent-child play and the emergence of behavior problems in childhood. To organize our results, we inferred four qualitative aspects of parental behavior: parental control and discipline, parental responsiveness and warmth, maternal sensitivity, affect and maternal depression. These four qualitative aspects were inductively inferred from key results of the final 39 articles that met all inclusion criteria while being geared to the two axes of parental behavior proposed by Maccoby and Martin (1983). Critical for including an article in a particular category was the naming of the investigated construct by the authors (e.g., maternal sensitivity) as well as the use of fitting measures [e.g., *Ainsworth's 9-point rating scale for Sensitivity and Cooperation* (Ainsworth et al., 1974) for maternal sensitivity]. In addition, we found studies that did not fit into one of the categories but related to more global aspects of interaction quality and quality of parent-child play or considered a specific variable that was not covered by any other study (mind-mindedness, teaching).

### Study Characteristics

The identified articles were published between 1982 and 2020 and all included studies had study samples from Western cultures, mainly North America (16 USA, 8 Canada) and Europe (1 Belgium, 3 Germany and Austria, 5 Netherlands, 5 United Kingdom), as well as one study each with participants from Israel and Australia. 19 of the selected articles described cross-sectional studies with a single point of data collection, while the remaining 20 studies collected data longitudinally over several points in time. All studies involved a play interaction between parent and child at home or in the laboratory, during which parental and—in some cases—child variables were collected. The length of this interaction varied between five minutes and three hours (Ø 27 mins) depending on the study and was recorded on video for coding in most cases (this was not the case in only six studies). In 82% of studies, the interaction between mothers and their children was investigated and only 8 studies examined both mothers and fathers or father-child interactions alone. While a range of different scales were used to code the parental variables in the interaction observation, the CBCL (as either parent or teacher-rating) was used to survey child behavior adjustment in 55% of the studies. Regarding children's behavioral adjustment, 9 studies focused on internalizing and 13 on externalizing behavior problems, while the remaining 17 articles considered both types of behavior problems. An overview of the data extracted from the reviewed articles can be found in Table 1 and further information on the individual studies can be found at https://osf.io/arg8j/.

## Narrative Synthesis

The included studies indicate that several parental variables during parent-child play (e.g., warmth, sensitivity, positive affect, responsiveness) are associated with reduced quantity and quality of externalizing and internalizing behavioral problems at the time of data acquisition or later on in childhood and adolescence. In contrast to that, inadequate or harsh control as well as negative affect and maternal depression correlated with more or more intense behavioral problems. Apart from those general results in line with our hypothesis and previous research, differences in methodology, samples, and concept definitions should be noticed.

### Parental Control and Discipline

Parental control is described as a crucial dimension of parenting behavior (Maccoby and Martin, 1983; Pinquart, 2017). In a range of studies identified in this literature review, parental control and discipline were observed during play interactions and found to show clear associations with concurrent or later child behavior problems. Five of those studies were observational and found a positive association between controlling and intrusive parenting patterns during parent-child play (e.g., parental harsh discipline, rejection) and more severe concurrent internalizing and externalizing behavior problems in children (Webster-Stratton and Eyberg, 1982; Denham et al., 1991; Mence et al., 2014; Dubois-Comtois et al., 2015; Janßen et al., 2019). Notably, all except one study correlated parenting patterns during play with behavioral adjustment whereas Janßen et al. (2019) compared a group of dyads with children with an ICD-diagnosis of externalizing or internalizing disorders to a control group without diagnosis. Yet, all studies found the same direction of association between parental control/discipline and children's behavioral adjustment, focusing on externalizing behavior problems. These results are further supported by four longitudinal studies investigating parent-child play in infancy and behavioral problems later in early childhood (Lee and Bates, 1985; Heller and Baker, 2000; Hughes and Ensor, 2007; Smeekens et al., 2007).

Despite their methodological differences, all these findings fit the results of preceding meta-analyses that found a negative association between negative parental control (e.g., harsh, excessive) and internalizing as well as externalizing behavior problems in children (McLeod et al., 2007a,b; Hoeve et al., 2009; Yap and Jorm, 2015; Pinquart, 2017). Interestingly, the results of Denham et al. (1991) and Webster-Stratton and Eyberg (1982) seem contradicting at first sight: Webster-Stratton and Eyberg (1982) found in a small sample of 35 mother-child dyads that maternal submissiveness during play (i.e., infrequent and ineffective use of parental control) was associated with behavior problems in children. Therefore, it seems that not only excessive control but also the opposite—submissiveness—is negatively associated with behavioral adjustment, what somehow contradicts Denham et al. (1991) findings that autonomy granting (also labeled as the opposite of control) is positively associated with behavioral adjustment. Yet, this contradiction can be solved by having a closer look at the definition of submissiveness and autonomy granting: submissiveness means no or nearly no (effective) use of control during play whereas autonomy granting is operationalized as encouraging the child to make own decisions and develop independence (Silk et al., 2003). Hence, these two concepts differ critically even though they look similar on first sight and the aforementioned findings do not contradict each other.

### Parental Responsiveness and Warmth

Besides the dimension of parental control, responsiveness and warmth of parents is considered the second crucial dimension of parenting behavior (Maccoby and Martin, 1983). In fact, most of the studies identified in the current literature review can be grouped to investigate this particular dimension, providing different results regarding whether parental warmth and/or responsiveness during play is associated with behavioral adjustment in children.

With respect to maternal responsiveness during play, a longitudinal study with a large sample of 424 dyads showed that children whose mothers were more sensitive and receptive in a play interaction were more likely to be assigned to a prosocial group than to an aggressive group (Jambon et al., 2019). Studies by Landy and Menna (2001) and Urbain-Gauthier and Wendland (2017) show similar results in relation to samples with aggressive children, revealing reduced maternal responsiveness and sensitivity in observed mother-child interactions compared to control groups with typically developing children. In addition, an observational study by Ambrose and Menna (2013) investigating interactional synchrony, supports these results. Importantly, however, longitudinal observations have failed to show protective effects of maternal responsiveness on the emergence of behavioral problems (Scaramella et al., 2008; Schmid et al., 2011; Sirois et al., 2019). Closely related to the concept of responsiveness and synchrony in mothers is the degree of warmth in mother-child interactions, which is often operationalized similarly to responsiveness. In studies focusing on maternal warmth, a similar pattern could be shown in that a lack of warmth as well as negative interaction with harsh parenting are associated with increased behavior problems in children (Hughes and Ensor, 2007; Scaramella et al., 2008; Schrock and Woodruff-Borden, 2010; Lorber and Egeland, 2011; Williams et al., 2012; Murphy et al., 2017). These findings are also consistent with preceding meta-analyses, that show only small positive effects for parental warmth on behavior adjustment if any and much larger effect sizes for the positive association of parental control or lack of warmth/responsiveness and children's behavioral problems (McLeod et al., 2007a,b; Khaleque, 2013; Pinquart, 2016, 2017). However, as described above for submissiveness and autonomy granting, it remains important to have a close look on how each study operationalizes warmth/responsiveness. Hoeve et al. (2009) found in their meta-analysis that it is important to differentiate whether the positive facets of the continuum (e.g., warmth, supportiveness, responsiveness) are measured in a study or the negative facets (e.g., hostility, rejection), as they found a quite larger association between negative facets and behavioral problems (delinquency) as for positive facets. This instance also fits the results of the studies included in the present narrative review (Scaramella et al., 2008; Schrock and Woodruff-Borden, 2010; Lorber and Egeland, 2011; Williams et al., 2012).

### Maternal Sensitivity

In the current literature review, 4 studies were identified that focused specifically on maternal sensitivity during play. Most of the studies used a longitudinal approach to correlate maternal sensitivity during free or semi-structured play interactions with children's internalizing behavioral problems (Morrell and Murray, 2003; NICHD Early Child Care Research Network, 2004; Kok et al., 2013; van der Voort et al., 2014). Several of these studies indicate that maternal sensitivity could be a possible mediator between a child's risk factor for developing behavior problems (inhibited behavior, poor affect-regulation skills) and the actual occurrence of behavior maladjustment in the future (Morrell and Murray, 2003; NICHD Early Child Care Research Network, 2004; van der Voort et al., 2014). Finding maternal sensitivity as a mediator or not, all studies revealed a consistent picture showing that maternal sensitivity observed during parent-child play seems to be related to behavioral adjustment in childhood. Nevertheless, it should be considered, that sensitivity is a broad concept, that includes different aspects from study to study und sometimes also includes facets of the aforementioned dimensions warmth/responsiveness and control (e.g., supportiveness, intrusiveness, attachment). For example, van der Voort et al. (2014) defined sensitivity as supportive presence, intrusiveness, sensitivity and timing, and clarity of instruction during play, whereas Kok et al. (2013) included cooperation, supportiveness, no intrusiveness, and consideration in their definition of maternal sensitivity. Yet, all these aspects are positively related to behavioral adjustment in children throughout literature (e.g., Jaffari-Bimmel et al., 2006; Roisman and Fraley, 2012).

### Affect and Maternal Depression

Consistently with results of previous meta-analyses (e.g., Beck, 1999; Goodman et al., 2011), 7 included articles show that maternal affect and emotion during parent-child play are associated with behavioral problems during and outside of the play interaction. For example, maternal positive emotions during play were associated with higher child socio-emotional competence (Denham et al., 1991) and fewer internalizing behavior problems (Robinson et al., 2009), while maternal anger (negative affect) was associated with more internalizing behavior problems (Dollberg et al., 2006; Robinson et al., 2009). This pattern is corroborated by another observational study, that investigated maternal affect during play in groups of social-competent, angry-aggressive and fearful-withdrawn children (LaFrenière and Dumas, 1992). Maternal depression is also closely associated with negative affect and emotion in mothers and therefore can affect parent-child play indirectly. Chronically dysphoric, depressive, or anxious mothers not only show more negative affect during mother-child play but also report more behavioral problems in their children, both in the internalizing and in the externalizing domain, as healthy mothers (Dumas et al., 1989; Lang et al., 1996; Endendijk et al., 2017). However, it should be noted that these results rely on self-report of depressed mothers, and it might be that this self-report is influenced by the maternal depression itself (Lang et al., 1996). Goodman et al. (2011) investigated this aspect in their meta-analysis and found, that both externalizing and internalizing behavior problems were rated significantly higher by depressive mothers than by preschool-teachers or the children themselves. Thus, it is crucial to consider how exactly a score of behavioral problems is determined before drawing conclusions about causality in such studies.

### Global and Specific Aspects of Parent-Child Interaction

Finally, 13 studies remain that either had a more global look on parent-child play or have investigated specific aspects that could not be grouped to the above categories of parental behavior. One such investigation is the study by Colonnesi et al. (2019), which focused in particular on parental mind-mindedness during free play. Mind-mindedness describes the extent to which parents recognize their children as individuals with their own thoughts and try to put themselves in their position and respond to them during play. In this study, mind-mindedness was assessed in a longitudinal design, showing that children's externalizing behavior problems were predicted by low levels of mind-mindedness in both parents. Another study specifically investigated the role of maternal teaching in mother-child interactions and found that more maternal teaching and child compliance during problem-solving tasks predicted better behavioral adjustment measured at a later time point (Lunkenheimer et al., 2013). Moreover, five studies investigated the quality of observed parent-child play and how this was associated with children's behavioral adjustment. In the majority of these studies, dyads of parents and children with externalizing problems were compared to non-disordered control dyads (Gardner, 1994; Landy and Menna, 2001; Gardner et al., 2003; Urbain-Gauthier and Wendland, 2017). These studies reveal that mothers of children with externalizing problems showed difficulties in supporting their child's pretend play and interrupted play more often (Urbain-Gauthier and Wendland, 2017) as well as showing less initiative in play and more difficulties keeping the play activity going (Gardner, 1994). Similar results were also shown for the positive association between higher quality of play and fewer internalizing behavior problems in children (Gardner et al., 2003; Ahnert et al., 2017). Additionally, a qualitative study by Landy and Menna (2001) examined mothers' behavior during a play interaction with aggressive content and found that mothers of aggressive children had greater difficulty in dealing with their children's aggressive play than mothers in the control group. The study revealed that mothers of aggressive children showed a reduced ability to intervene sensitively and thereby to lead the play in a more positive, conciliatory direction (e.g., in reaction to the fact that “the lion eats the crocodile,” to reconcile all the animals with each other again in a joint “excursion to the park”) (Landy and Menna, 2001). This qualitative study provides an important insight into which parental behaviors in detail lead to higher or lower quality of play and complements quantitative results with vivid examples.

Finally, some studies have grouped several aspects of interaction into a general variable describing the quality of parent-child interaction during play (Gardner, 1987; Yates et al., 2010; Dubois-Comtois et al., 2015; Cao et al., 2020). Similar to the broad concept of parental sensitivity, these studies differed in their definition of interaction quality: for example, Dubois-Comtois et al. (2015) observed coordination, communication, emotional expression, responsivity/sensitivity, tension, mood, and enjoyment to determine interaction quality, whereas Cao et al. (2020) measured global maternal positivities and negativities including aforementioned concepts as sensitivity, intrusiveness, supportiveness, autonomy granting, and hostility. Nevertheless, all these studies found a positive association between quality of parent-child play and fewer externalizing as well as internalizing behavior problems (Gardner, 1987; Dubois-Comtois et al., 2015; Cao et al., 2020). Notably, Yates et al. (2010) solely found cross-sectional correlations between interaction quality and behavior problems but no longitudinal associations in their longitudinal study, despite other studies have found that certain parental variables predict children's behavior problems at a later time point (e.g., Denham et al., 1991; Scaramella et al., 2008; Lorber and Egeland, 2011; Ambrose and Menna, 2013; Dubois-Comtois et al., 2015; Murphy et al., 2017; Janßen et al., 2019). Nevertheless, this mixed evidence is also reflected in recent meta-analyses, that point out that direction and causality of the associations between parental variables and behavior problems are not sufficiently investigated yet (McLeod et al., 2007a,b; Pinquart, 2017).

### Study Quality

The included studies were all analyzed concerning power, effect sizes, and strong methodological issues (e.g., small group sizes) (see supplemental information at https://osf.io/arg8j/). In this regard, three methodological points were notable:

Firstly, the instruments measuring parental variables differed widely across the included studies. Respective authors state, that they were all validated in previous research, but, nevertheless, comparability of results between studies is therefore limited and the establishment of a comprehensive instrument for parental variables during parent-child play is eligible. A second factor of methodological variety is the design, namely some studies comparing groups of children with diagnosed behavioral problems with groups of children without these problems, and some studies correlating parental variables and children's behavioral problem scores. A meta-analysis by McLeod et al. (2007a) indicates, that in general, studies comparing groups of children with diagnosed behavioral problems to controls achieve higher effect sizes than studies correlating behavioral problem scores with parental variables. Yet, the exact effect sizes are not essential to a narrative synthesis as we did. Finally, there were some studies that used small samples in their group comparison (e.g., Gardner, 1987: 20 children with conduct problems vs. 19 controls), that should be considered interpreting their results. However, all these studies found similar results as studies with larger samples and effects sizes. All in all, it should be noted that these methodological varieties across studies strengthen the conclusions of this review, as most studies found the same associations despite their different methodological approaches.

## Summary and Conclusion

This systematic literature search sought to shed more light on the role of parent-child play in the emergence of behavioral adjustment. Specifically, we asked how parental behavior characteristics during parent-child play in early childhood might be related to the emergence of behavioral adjustment and the onset of externalizing and internalizing behavioral problems. Following a preregistered procedure, we identified 39 articles that represented four dimensions of parental behavior characteristics during play: parental control and discipline, parental responsiveness and warmth, maternal sensitivity, and affect and maternal depression. In addition, we found studies that investigated more global aspects of parent-child interactions as well as specific aspects that did not fit the aforementioned categories (e.g., quality of play). Several included studies reveal that increased restrictive and inappropriate control during play interactions was associated with increased externalizing behavior problems (but see Webster-Stratton and Eyberg, 1982). Similarly, a lack of parental responsiveness and warmth were found to be associated with increased behavioral problems, although no protective effect could be found in the context of risk groups. Consistent with this view, a lack of maternal sensitivity during parent-child play was found to be related with increased behavior problems. Other studies indicated that positive affect expressed by parents during parent-child play was associated with fewer behavior problems in children, while negative affect—often in the context of maternal depression—was associated with more behavior problems. Finally, our review revealed that the quality of play differs significantly between groups of children with and without behavioral problems, with reduced quality and quantity of play in children with difficulties in behavioral adjustment. Additionally, the general quality of interaction was positively associated with less behavioral problems.

In sum, these range of findings suggest that the play situation might constitute an important window in which parents exert influence on their children's social and emotional development. Our findings show that certain parental behavior characteristics during parent-child play might affect the child's development of internalizing and externalizing behavior problems. As hypothesized, positive parenting variables as warmth, responsiveness and sensitivity were associated with fewer behavioral problems whereas negative parenting variables as harsh control or negative affect correlated with more behavioral problems across different study settings and samples.

## Limitations and Future Perspectives

Some limitations should be noted when considering the findings of the present review. First, although we aimed to include studies specifically investigating parental playfulness, our systematic literature search did not bring up such studies. This could be due to two possible factors: (1) the syntax should have included additional specific keywords like *rough-and-tumble play* or *withdrawal*, (2) up to April 2021, there were no (or only few) studies that explicitly investigated the association between parental playfulness and behavioral adjustment of children. Second, from the included studies no inference about causality can be drawn, i.e., if certain parental interaction characteristics lead to more behavioral problems in children or if certain behavioral problems lead to certain parental interaction characteristics. Nevertheless, longitudinal studies, which show that certain parent behavior predicts later behavior adjustment of children (e.g., Gardner et al., 2003; Hughes and Ensor, 2007; van der Voort et al., 2014) provide some insight into the directionality of the observed effects. Finally, it should be noted that some minor adjustments of the original preregistration were necessary. As an additional step after title and abstract screening, we excluded all papers that examined PCIT or similar interventions since interventions were not the focus of this review (which is why we clearly excluded *play therapy* in the preregistered syntax) and would go beyond our research question. Moreover, the considered methodological checklist by Downs and Black (1998) was not suitable to check the methodological quality of the included studies and studies were analyzed concerning power, effect sizes, and strong methodological issues (e.g., small group sizes) instead (see supplemental information at https://osf.io/arg8j/).

In the light of these limitations, future studies should consider directly investigating the impact of parental playfulness on children's behavioral adjustment. Although clinical applications already make use of the play situation, such as through parent training (e.g., PCIT, Eyberg et al., 1995), in order to foster mental health and behavioral adjustment in children, empirical research has investigated this association mostly in an indirect way. It remains mostly unexplored how parent-child play itself and parental playfulness contribute to behavioral adjustment, even though not only Erikson's model but also recent research indicate an association between parental playfulness and child characteristics (e.g., emotion regulation; Shorer et al., 2019). Since parental playfulness seems to be closely linked to parental behavior characteristics like warmth, control, and sensitivity, that in turn are positively related to behavioral adjustment (Lucassen et al., 2011; Waller et al., 2015), the specific investigation of parental playfulness is a promising aspect for future research on the factors affecting the development of child behavioral problems. Especially, future studies using longitudinal designs to investigate parental playfulness as well as children's behavioral adjustment each at several time points across children's development are highly eligible. Finally, the vast majority of studies included in this review investigated parental characteristics of mothers. This reflects another important gap in the literature and starting point for future research, since differences between mothers' and fathers' play with their children have been identified, that might also contribute differentially to child development (Cabrera et al., 2017).

## Data Availability Statement

The original contributions presented in the study are included in the article/supplementary material, further inquiries can be directed to the corresponding author/s.

## Author Contributions

PB conceived the research. MS, PB, and IF conducted the research. MS and PB wrote the first draft of the paper. IF edited the article. All authors contributed to the article and approved the submitted version.

## Funding

Open Access article charges are covered by the Max Planck Digital Library (MPDL).

## Conflict of Interest

The authors declare that the research was conducted in the absence of any commercial or financial relationships that could be construed as a potential conflict of interest.

## Publisher's Note

All claims expressed in this article are solely those of the authors and do not necessarily represent those of their affiliated organizations, or those of the publisher, the editors and the reviewers. Any product that may be evaluated in this article, or claim that may be made by its manufacturer, is not guaranteed or endorsed by the publisher.
